# Comparison of DGT with traditional methods for assessing cadmium bioavailability to *Brassica chinensis* in different soils

**DOI:** 10.1038/s41598-017-13820-3

**Published:** 2017-10-27

**Authors:** Yunchao Dai, Mubasher Nasir, Yulin Zhang, Haiming Wu, Honghong Guo, Jialong Lv

**Affiliations:** 1 College of Natural Resources and Environment, Northwest A&F University, Yangling, 712100 China; 2 Key Laboratory of Plant Nutrition and Agri-environment in Northwest China, Ministry of Agriculture, Yangling, 712100 China

## Abstract

There is no universally accepted method for evaluating cadmium (Cd) bioavailability in soil. The diffusive gradient in thin films (DGT) technique is a promising tool, but there is considerable debate about its suitability. The ability of this technique to estimate Cd bioavailability in soils was compared with the abilities of other traditional chemical extraction techniques (soil solution, ethylene diamine tetraacetic acid (EDTA), acetic acid (HAc), calcium chloride (CaCl_2_), and pseudo-total Cd methods) based on a greenhouse experiment using pakchoi (*Brassica chinensis*) grown in 15 soils from different provinces of China. In addition, we assessed whether these methods were independent of the soil properties. Correlations between the plant and soil Cd concentrations measured with the traditional extraction techniques were dependent on the pH and organic carbon (OC) content, indicating that these methods are influenced by the soil properties. In contrast, the DGT measurements were independent of the soil properties and showed a higher correlation coefficient compared to that of the traditional techniques. Hence, the DGT technique is better and should be preferable for assessing Cd biological effectiveness in different soil types.

## Introduction

Cadmium (Cd) is a highly toxic heavy metal due to its long-term persistence and permanent impact on organisms. Many studies have reported that Cd is highly soluble in soil and can be easily taken up by plants, which further increases its risk to humans^1–3^. Existing regulations for Cd in soils are generally based on total concentrations, but such measures do not reflect the biological effectiveness of this pollutant^4^. Thus, there is no universally accepted approach for evaluating Cd bioavailability in soil.

Traditional methods for evaluating Cd bioavailability in soil include the isotope dilution exchange method^5^, sequential extraction^6,7^, and the free metal ion activity model^8,9^. Single-step chemical extractions include the use of chelating agents such as ethylene diamine tetraacetic acid (EDTA), acids such as acetic acid (HAc), and neutral salts such as calcium chloride (CaCl_2_)^10^. Single-step chemical extraction methods are simple and convenient. However, these equilibrium-based methods have disadvantages, as they do not consider root uptake at the root–soil boundary. For example, it is difficult to prevent re-adsorption of metals to the solid soil phase during chemical extraction, which may negatively impact the results^11–13^. It is still not clear whether free ion activity in soil solutions can indicate the biological effectiveness of heavy metal in soils^14–17^.

Generally, metal absorption by plants is related to dynamic interactions between the solid phase, soil solution, and roots. Recently, the DGT technique has been introduced as a tool for assessing the bioavailability of metals in soil^18–20^. The principle of DGT is based on Fick’s first law of diffusion. A concentration gradient is established towards the resin gel, and the DGT acts as “zero sink” for solutes. Elements diffuse through a diffusive gel and are bound by a resin gel, resulting in a lower concentration of the target element at the soil-DGT interface. Thus, more elements are desorbed from the soil solid phase and diffuse through the DGT surface. The process of element desorption from the soil solid phase and diffusion through the DGT surface is similar to the uptake of an element by plants^21–23^.

Various soil properties, such as pH, cation exchange capacity (CEC), clay content, and organic carbon (OC) content, affect the Cd taken up by plants^24^. When exploring how soil properties influence the ability of a method to predict Cd bioavailability, it is important to consider the soil properties, bioavailable Cd concentrations and Cd accumulation in plants.

Linear relationships between soil and plant Cd concentrations measured by various typical analytic methods are used to predict the biological effectiveness of Cd. However, single factor correlation analysis cannot evaluate the impact of the soil properties on Cd absorption. In this study, both simple linear regressions and stepwise multiple linear regressions (SMLRs) were applied to explore the correlations between soil properties and Cd uptake by pakchoi (*Brassica chinensis*). In light of the serious contamination of leafy vegetables, *Brassica chinensis* was selected as a test vegetable crop due to its high biomass even when grown in Cd-contaminated soils^25,26^.

The main goals of this work were to compare different methods for assessing Cd bioavailability in soils with diverse physicochemical properties, investigate the effectiveness of these techniques in assessing Cd uptake by *Brassica chinensis*, and evaluate the influence of soil properties on the outcomes of the tested methods.

## Results

### Plant biomass in different soil types

**Figure 1:**
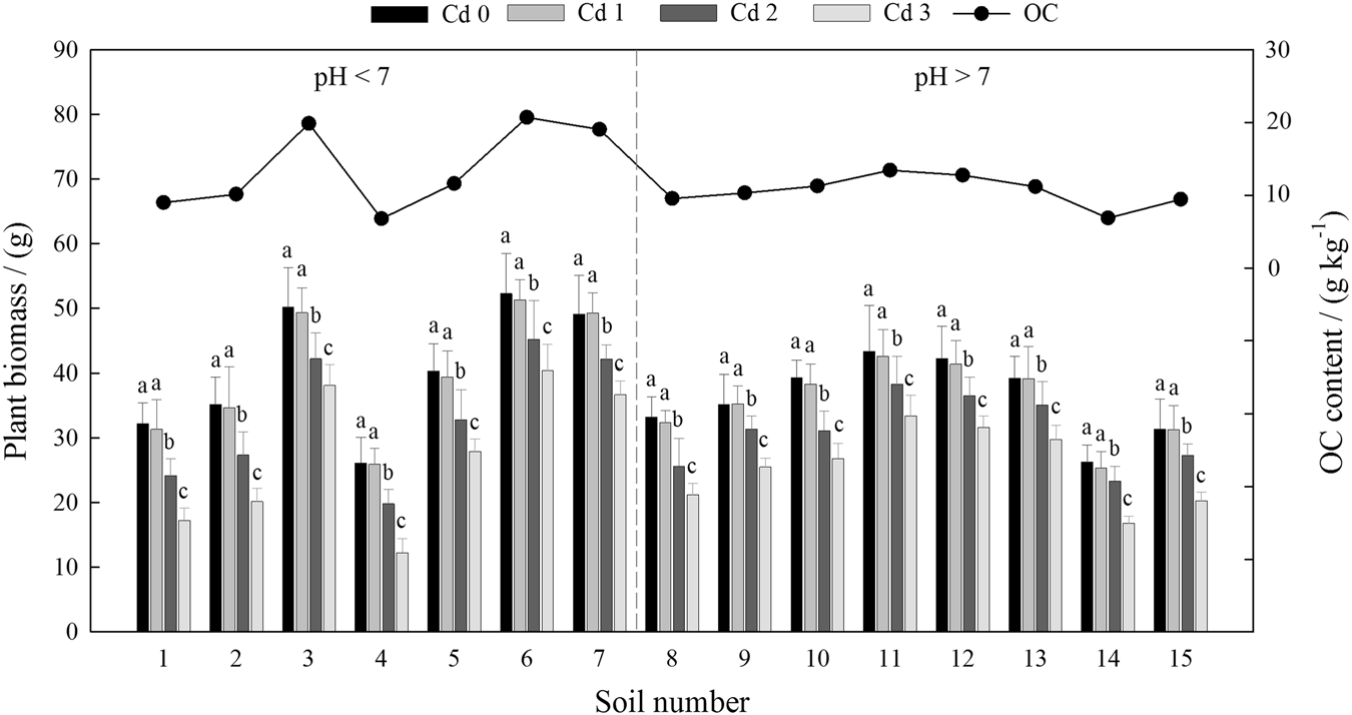
Comparison of the plant biomass in 15 different soils. The soils are listed in order of increasing pH. Bars indicate the standard deviation (n = 3). The different letters within each soil indicate significant differences at *P* < 0.05.

The soils used in the experiment varied with respect to pH (4.90–8.80), CEC (8.70–31.11 cmol·kg^−1^) and OC (6.78–20.70 g·kg^*−*1^). The average plant biomass in the different soils was 33.72 ± 3.38 g (Fig. 1). The average plant biomasses observed in the Cd 0, Cd 1, Cd 2, and Cd 3 treated samples were 38.38 ± 4.46 g, 37.79 ± 3.62 g, 32.17 ± 3.28 g, and 26.55 ± 2.17 g, respectively. The average plant biomass in Cd 1 was not significantly different from that in Cd 0. The plant biomasses in Cd 2 and Cd 3 were significantly lower in all soils relative to those in Cd 0 and Cd 1 (*P* < 0.05); this may be due to the low tolerance of plants to high Cd concentrations. In addition, the plant biomass observed in Cd 2 was lower in the acidic soils than in alkaline soils. However, the reduction in plant biomass was not significant in alkaline soils, possibly due to the stronger buffering capacity of alkaline soil to Cd contamination.

### Cd accumulation in plants

**Figure 2:**
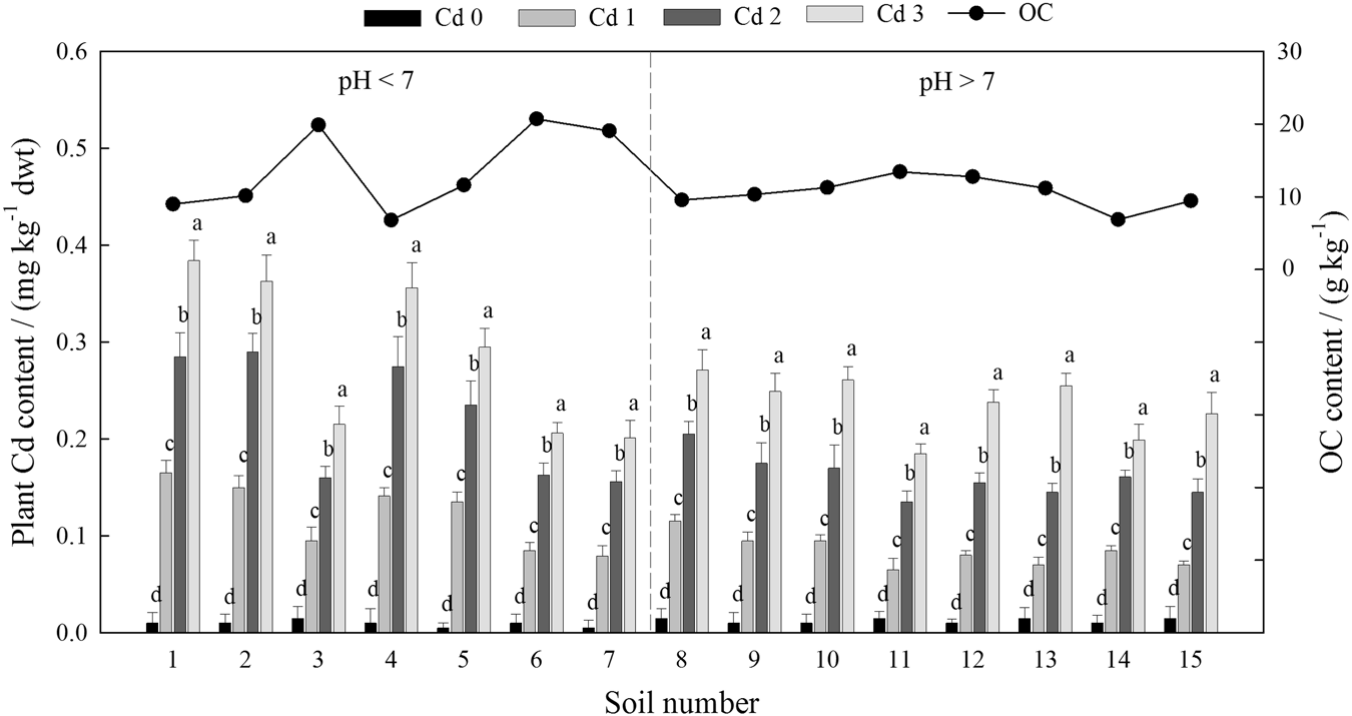
Cd content absorbed by plants grown in the 15 different soils. The soils are listed in order of increasing pH. Bars indicate the standard deviation (n = 3). The different letters within each soil indicate significant differences at *P* < 0.05. dwt indicates dry weight basis.

The average plant Cd concentration was 0.141 mg·kg^−1^ in all soils. The Cd concentration in the plants dramatically increased with increasing concentration of Cd in all soils. The Cd contents in soils 1, 2, 4, and 5 were significantly higher than the average value (Fig. 2). The total plant Cd contents in Cd 0, Cd 1, Cd 2, and Cd 3 ranged from 0.005 to 0.015 mg·kg^−1^ (average: 0.011 ± 0.009 mg·kg^−1^), 0.065 to 0.165 mg·kg^−1^ (average: 0.102 ± 0.009 mg·kg^−1^), 0.135 to 0.290 mg·kg^−1^ (average: 0.190 ± 0.016 mg·kg^−1^), and 0.185 to 0.384 mg·kg^−1^ (average: 0.260 ± 0.018 mg·kg^−1^), respectively. The plant Cd contents in Cd 1, Cd 2, and Cd 3 were significantly higher than that in Cd 0 for all soils (*P* < 0.05). The average plant Cd contents in acidic soils (1–7, pH 4.90–6.82) and alkaline soils (8–15, pH 7.90–8.80) were 0.161 and 0.124 mg·kg^−1^, respectively, presumably due to the lower Cd bioavailability in alkaline soils. This indicates that a low pH can enhance the bioavailability of Cd in soils. However, the Cd content in plants grown in acidic soil 3 (0.121 mg·kg^−1^) was significantly lower than the average value (0.161 mg·kg^−1^), probably due to the higher OC content in this soil (19.87 g·kg^−1^), which increased Cd absorption to the soil. This indicates that OC can also immobilize Cd in soils and decrease its bioavailability.

### Relationships between plant and soil Cd contents

**Figure 3:**
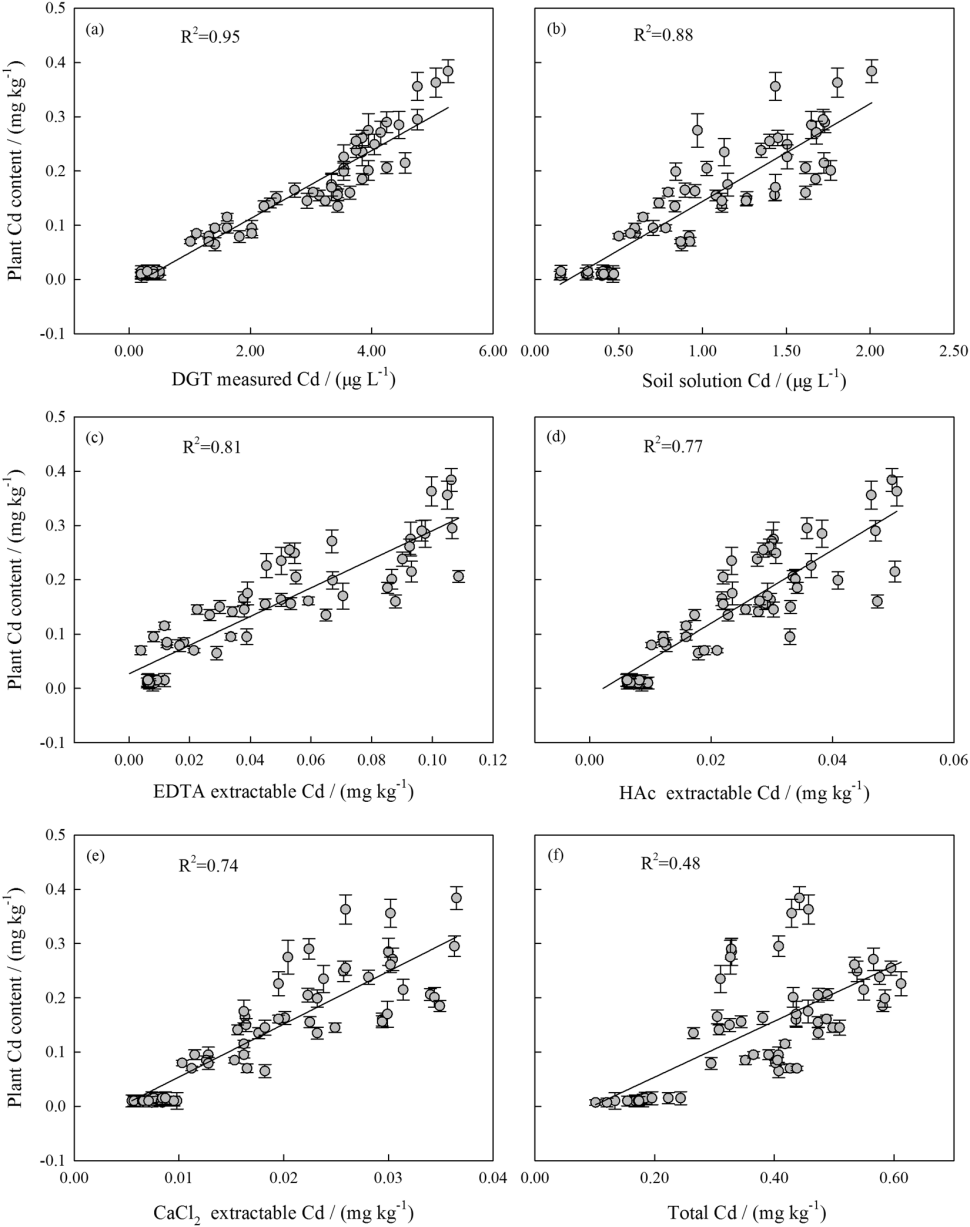
Relationships between plant and soil Cd contents. Bars indicate the standard deviation (n = 3).

The relationships between Cd concentrations in *Brassica chinensis* and the soil Cd concentrations measured with DGT, soil solution, EDTA, HAc, CaCl_2_, and pseudo-total Cd methods are shown in Fig. 3. The concentrations measured with both the DGT and soil solution methods were positively correlated to the plant Cd concentration, but the correlation coefficient for DGT was higher. The correlation coefficients for the linear regressions decreased in the following sequence: DGT method > soil solution method > EDTA method > HAc method > CaCl_2_ method > pseudo-total Cd method. These extraction methods varied in their ability to assess the Cd bioavailability. Based on these results, the DGT technique is the best method for predicting Cd bioavailability to uptake by *Brassica chinensis* grown on different soils.

### Stepwise multiple linear regressions

For the SMLRs, the soil properties and the Cd concentration in soil were selected as independent variables, and the plant Cd concentration was selected as the dependent variable. Different regression analyses were performed for the soil Cd concentrations measured with different methods (DGT, soil solution, EDTA, HAc, CaCl_2_, and soil pseudo-total Cd methods), and their prediction equations are shown in Table 1.

**Table 1:** Prediction equations calculated by stepwise multiple linear regressions (n = 60)^a^.

Eq. No.	Prediction Eq.	*R* ^2^
2	Plant-Cd = 0.064 DGT-Cd - 0.008 pH - 0.015	0.95**
3	Plant-Cd = 0.183 soil solution-Cd – 0.005OC – 0.013 pH + 0.114	0.92**
4	Plant-Cd = 2.679 EDTA-Cd – 0.003 OC + 0.072	0.89**
5	Plant-Cd = 7.443 HAc-Cd – 0.014 pH – 0.004OC + 0.136	0.84**
6	Plant-Cd = 9.979 CaCl_2_-Cd – 0.006OC – 0.017 pH + 0.149	0.82**
7	Plant-Cd = 0.356 total-Cd - 0.034 pH - 0.005OC + 0.350	0.78*

*Correlation is significant at *P* < 0.05, **correlation is significant at *P* < 0.01. ^a^Five soil properties, *i.e*., pH, OC, CEC, CaCO_3_ and clay content, were used as inputs, and plant Cd concentrations were used as outputs. The significance level was *P* > 0.05 for CEC, CaCO_3_, and clay content in each equation and *P* < 0.01 for pH and OC in each equation except for Eq. 2 (*P* < 0.05 for pH in Eq. 2).

The soil pH and OC were negatively correlated to the Cd bioavailability. These soil properties greatly influenced the strength of the regressions for all the traditional extraction methods, whereas the*R* ^2^ value for the DGT method remained almost the same (Table 2). Moreover, pH and OC were significant variables in the regressions for the soil solution, EDTA, HAc, CaCl_2_, and pseudo-total Cd methods, and the *R* ^2^ values improved from 0.88 to 0.92, 0.81 to 0.89, 0.77 to 0.84, 0.74 to 0.82, and 0.48 to 0.78, respectively.

**Table 2:** Regression coefficients (*R* ^2^) for the linear regressions and stepwise multiple linear regressions (n = 60).

	DGT	Soil solution	EDTA	HAc	CaCl_2_	Pseudo-total Cd method
Linear regressions	0.95	0.88	0.81	0.77	0.74	0.48
Multiple regressions	0.95	0.92	0.89	0.84	0.82	0.78

Similar to the simple linear regression results, the correlation coefficients for the SMLRs decreased in the following sequence: DGT method > soil solution method > EDTA method > HAc method > CaCl_2_ method > pseudo-total Cd method. This confirms that the DGT technique is superior to the traditional techniques for evaluating the Cd bioavailability.

## Discussion

Several studies have reported that the soil pH and OC content greatly affect Cd bioavailability in soil solutions as well as the distribution of Cd in liquid and solid phases^27^. Acidic soils have a weak Cd buffering capacity, allowing for Cd mobilization; therefore, plants grown in such soils are prone to Cd uptake from the soil solution. In the current study, the Cd contents of plants grown in acidic soils mostly exceeded the toxic threshold due to uptake by the roots (Fig. 2). A previous study^28^ reported that rhizospheric soil contains low-molecular-weight organic acids, which can easily dissolve Cd and promote Cd uptake by plant roots. Soil OC has been reported to immobilize Cd in soil; therefore, Cd toxicity is lower in highly alkaline soils with a high OC content^29^.

Based on the regressions for the various methods investigated in this study, the prediction accuracy of Cd bioavailability was in the following sequence: DGT method > soil solution method > EDTA method > HAc method > CaCl_2_ method > pseudo-total Cd method. These results indicate that the dominant supply processes in these soils are diffusion and labile metal release. Local decreases in the soil solution concentration induce desorption of the metal supply from the solid phase, as shown by a dynamic model of the DGT soil system^30^. A concentration gradient can be established at the soil-sampler interface using DGT, unlike in traditional soil extraction methods, which are mainly based on equilibrium concentrations related to the molarity of the extraction agent. The soil solution method extracts the water-soluble form and some of the exchangeable forms of metals, which are the main forms taken up by plants. EDTA has a high extractant capability; it can extract water-soluble Cd, exchangeable Cd, iron and manganese oxides combined with Cd, and organic matter–Cd complexes. HAc is an acidic extractant that can extract water-soluble Cd, exchangeable Cd, and carbonate-bound Cd. CaCl_2_ is a neutral salt extractant that can only extract exchangeable metals^31^. The pseudo-total Cd method measures fractions that are unavailable to plants. Therefore, the kinetically labile metal in the solid phase clearly plays an important role in plant uptake and is included in the DGT measurement. Traditional methods do not fully reflect the solid-phase supply processes in soils and the re-adsorption of metals during the extraction process. Though the DGT method has obvious merits, some studies have proposed that the predictive capabilities of DGT depend on the concentration ranges of the metals in the soils as well as the plant species^5^. For example, in a study on the bioavailability to lettuce (*Lactuva sativa cv Appia*) in nine soils polluted with Zn and Cd, Cornu *et al*.^32^ found that plant Cd concentrations were weakly related to the DGT-based Cd concentrations. Koster *et al*.^33^ reported that the DGT method does not perform well in predicting Zn uptake by grass, lettuce, and lupine in terrestrial ecosystems.

Both simple linear regressions and stepwise multiple linear regressions showed that the DGT technique provided a better assessment of Cd bioavailability than the traditional extraction methods^34^. Soil parameters may impact Cd bioavailability^35^. The relationship between the plant and soil Cd contents that were evaluated with traditional extraction methods was relatively weaker than that determined by the DGT method. Traditional extraction methods do not always consider the effects of the soil characteristics because they do not include kinetic metal absorption by plants, and thus, they only reflect the equilibrium state of soil metals^36^. We showed that the traditional extraction methods are sensitive to soil pH and OC content, which is similar to the results of previous studies^37,38^. Thus, the soil properties should be considered when these traditional extraction methods are applied in the evaluation of Cd bioavailability.

The DGT technique includes contributions from the liquid and solid phases of soil as well as the exchange dynamics between the two phases^39^. The dynamic exchange of Cd from the solid to the liquid phase is an important factor that influences Cd uptake, and the Cd concentrations measured with DGT reflect these processes. The Cd concentration was locally reduced close to the surface of the DGT device, causing a gradient in the soil solution and diffusive gel. This produced a flux from the solid to the liquid phase, which facilitated natural depletion. These basic processes are identical to the metal uptake mechanisms by roots in soil^40^. Previous *in situ* studies have shown that the DGT method is better than the traditional extraction methods for predicting Cu, Cd, Zn, and Pb absorption by rice in field experiments^36^. In addition, DGT has been successfully used to assess the metal concentrations in plants grown in pot experiments^19,32^, and the measured concentrations were linearly correlated with increasing concentrations of metals in the soil^41^. Thus, the DGT technique has potential for evaluating metal bioavailability in both pot and field experiments. Although greenhouse experiments have some limitations, it is easier to control the environmental conditions, such as temperature and humidity, and several studies have reported that the results of greenhouse experiments can be verified by field tests^42^.

Several studies have concluded that the DGT technique is superior to traditional methods for assessing Cd bioavailability in different species of plants, including wheat, maize^43^, and ryegrass^44^. Although heavy metal contamination of leafy vegetables is a common and serious problem^25,26^, there is lack of relevant studies related to Cd bioavailability. In this work, a leafy vegetable (*Brassica chinensis*) was selected as the test plant because its biomass is not significantly affected by Cd pollution. Our results provide reliable and relevant evidence for the use of DGT to assess Cd bioavailability.

We showed that a simple empirical model that includes soil pH, OC, and bioavailable Cd content can be used to assess the performance of traditional extraction methods. The DGT technique was better than the traditional extraction methods for predicting Cd bioavailability because it includes the effects of soil properties as well as exchange dynamics between the solid and liquid phases. Therefore, DGT is a promising technique for assessing Cd bioavailability, as soil properties do not need to be further considered when using this method.

## Conclusions

The plant Cd concentrations increased significantly upon increasing the dose of Cd in all experimental soils. The Cd concentrations in plants grown in acidic soil were the highest due to the low buffering capacity of soil under acidic conditions. A linear correlation analysis revealed that the DGT technique was better than the conventional techniques for evaluating Cd bioavailability. The abilities of the tested methods to predict Cd bioavailability declined in the following order: DGT method > soil solution method > EDTA method > HAc method > CaCl_2_ method > pseudo-total Cd method. The correlations between plant and soil Cd concentrations evaluated with the traditional techniques were pH and OC dependent. Thus, soil pH and OC should be considered when using traditional techniques to assess the bioavailability of Cd in soils. DGT measurements are independent of the soil properties and thus are preferable for assessing Cd bioavailability.

## Materials and Methods

### Soil samples

Fifteen different top soil samples were collected throughout China. The soil samples varied in their physicochemical properties; pH and OC showed especially high variations (Table 3). Samples were taken from the top 0–20 cm of soil, air dried at 20 °C, and passed through a 2-mm sieve before measurement of the physicochemical properties.

**Table 3:** Physicochemical properties and background concentrations of metals in the different soils.

Soil No.	Location	Coordinates	pH	OC (g·kg^−1^)	CaCO_3_ (g·kg^−1^)	CEC (cmol·kg^−1^)	Clay (%)	Background Cd (mg·kg^−1^)	Background Pb (mg·kg^−1^)	Background Cr (mg·kg^−1^)	Background As (mg·kg^−1^)	Background Hg (mg·kg^−1^)	Soil quality^a^
1	Hunan	26°45′ N, 110°52′ E	4.90	9.00	0.00	10.85	42.91	0.19	28.74	49.69	16.59	0.08	I
2	Chongqing	29°48′ N, 106°24′ E	5.74	10.14	0.00	21.34	24.96	0.20	38.46	31.01	4.15	0.06	II
3	Yunnan	24°52′ N, 102°49′ E	5.92	19.87	0.00	11.10	27.52	0.30	42.51	53.74	8.71	0.09	II
4	Jiangxi	28°12′ N, 116°56′ E	6.01	6.78	0.00	8.70	36.51	0.18	34.75	44.81	11.64	0.07	I
5	Anhui	31°55′ N, 117°11′ E	6.25	11.62	0.00	19.08	16.84	0.11	30.17	36.96	8.34	0.07	I
6	Heilongjiang	45°40′ N, 126°37′ E	6.27	20.70	0.00	28.59	19.33	0.24	44.79	54.22	9.03	0.06	II
7	Jilin	43°31′ N, 124°48′ E	6.82	19.05	0.00	31.11	30.18	0.14	44.73	35.07	11.13	0.08	II
8	Shaanxi	34° 17′ N, 108° 01′ E	7.90	9.56	35.60	22.37	26.01	0.24	37.40	58.27	13.44	0.09	II
9	Henan	35° 00′ N, 113° 41′ E	8.07	10.32	27.50	16.01	18.18	0.23	36.12	50.97	9.22	0.09	II
10	Xinjiang	43°56′ N, 87°16′ E	8.12	11.27	15.06	25.25	9.57	0.20	40.87	73.42	10.82	0.07	II
11	Shanxi	37°22′ N, 112°28′ E	8.24	13.44	25.15	16.80	17.74	0.23	38.58	52.78	9.75	0.10	II
12	Tianjin	38°45′ N, 117°06′ E	8.29	12.77	53.57	24.67	7.59	0.22	39.75	46.26	12.26	0.06	II
13	Gansu	38°52′ N, 100°26′ E	8.37	11.18	38.51	11.23	6.66	0.21	36.86	79.17	13.94	0.09	II
14	Shandong	37°27′ N, 116°30′ E	8.65	6.87	31.69	13.09	17.11	0.26	36.02	59.86	8.29	0.07	II
15	Inner Mongolia	41°33′ N, 110°01′ E	8.80	9.45	11.51	11.61	10.51	0.22	40.80	66.00	7.24	0.07	II

^a^Classification of soil quality based on GB15618-1995 (China). Grade I: natural background level; Grade II: lightly polluted, safe for agricultural production and human health.

Soil analysis was conducted before aging the soil by following a previous protocol^29^. Briefly, the soil pH was determined with a pH meter (FE 20, Mettler Toledo, Shanghai, China) at a soil:water ratio of 1:2.5 w/v. The organic carbon (OC) content was determined with a mixed solution of K_2_Cr_2_O_7_ (0.4 mol·L^−1^) and H_2_SO_4_ (18 mol·L^−1^) using an oil bath heating method. CEC was calculated with the NH_4_OAc washing method. Briefly, the soil samples were washed three times with sodium acetate (NaOAc) to replace the cations. Then, the soil samples were washed with ammonium acetate (NH_4_OAc) to bring Na^+^ into solution, and finally, the Na^+^ concentration in the supernatant was measured using a flame photometer (FP 6410, Shanghai, China). The soil clay content was determined with the standard pipette method, and the calcium carbonate (CaCO_3_) content was measured with the gasometric method^45^. The total background concentrations of Cd, lead (Pb), and chromium (Cr) in soil were extracted using wet acid digestion [HNO_3_ (9.8 mol·L^−1^)-HF (27.05 mol·L^−1^)] in a sealed microwave digestion system^45^, and their concentrations were determined by atomic absorption spectrometry (AAS; Hitachi z-2000, Japan). The detection limit and precision of AAS were 6 μg·L^−1^ and 4 μg·L^−1^, respectively. Soil arsenic (As) and mercury (Hg) were extracted using aqua regia digestion according to the guidelines of the “Soil Quality-Analysis of Total As and Hg Contents in Soils” (GB/T 22105.1–2008). The total As and Hg concentrations in the soil samples were determined via atomic fluorescence spectrometry (AFS-930, Jitian, Beijing, China). The detection limit of AFS was 0.01 μg·L^−1^. The soil physicochemical properties and background metal concentrations are given in Table 3.

### Pot experiment and plant analysis

A greenhouse pot experiment was conducted in the Yangling Area of China. Cd was applied in the form of 3CdSO_4_·8H_2_O (AR, Bodi, China) to each pot (2 kg of soil) at concentrations of 0, 0.3, 0.45, and 0.6 mg·kg^−1^ (Cd 0, Cd 1, Cd 2, and Cd 3). After dissolution in water, 3CdSO_4_·8H_2_O was sprayed onto the soil, and the soil was evenly mixed. The soil was not mixed manually during the incubation period. The pots were incubated for 90 consecutive days, during which the soil water content was maintained at 75% of the maximum water-holding capacity (MWHC) using distilled water^46^.

After aging, each pot was supplemented with basic N, P, and K fertilizers in the form of urea, calcium phosphate, and potassium sulfate (0.3, 0.1, and 0.2 g), respectively. Five *Brassica chinensis* seeds were sown in the treated soils on August 1, 2015. The number of seedlings per pot was reduced to two plants after germination. Each treatment was replicated three times, and the pots were randomly arranged. The plants were grown under 14 h/10 h day/night and 20 °C/16 °C day/night temperatures. The soil moisture content was kept at 80% of the MWHC during the plant growth period.

Mature plants were harvested after 60 days. Fresh plant samples were thoroughly washed with distilled water, oven dried at 100 °C, and digested with HNO_3_ (9.8 mol·L^−1^) - H_2_O_2_ (16 mol·L^−1^) in a sealed high-pressure system. The total Cd concentrations in the plant samples were measured with AAS (Hitachi z-2000, Japan). The soil was collected after harvesting, air dried at 20 °C and passed through a 2-mm sieve for DGT and single-step extraction measurements.

Each treatment was replicated three times. GBW 10015 and GBW 07408 were used as standard reference materials for the plant and soil samples, respectively, to guarantee the accuracy of the tests. The standard samples were used for each batch of samples, and the recovery ratios ranged from 94% to 103% for the plant samples and from 96% to 104% for the soil samples throughout the analysis procedure.

### Analytical methods for measuring Cd bioavailability in soil

#### DGT measurement

Piston-type DGT devices were purchased from Weishen DGT Research Ltd., (Nanjing, China). Chelex-100 was used to prepare the resin gel for Cd binding. Details of the DGT device have been reported in a previous study^10^. The soil moisture strongly influences the performance of DGT for measuring metals in soils. For the DGT device to work properly, the soil moisture should be kept at or above 80% field capacity^47^. The devices were installed in the soils following standard procedures^39,48^. Subsamples were first collected for MWHC measurements. Fifty grams of air-dried soil was maintained at 60% MWHC for 48 h and then at 80% MWHC for the next 24 h. Then, the DGT devices were inserted into the soil surface, ensuring complete contact with the soil. The temperature was kept at 20 °C ± 1 °C during the deployment period. After 24 h, all DGT devices were retrieved from the soil, washed with distilled water to remove adhering soil, and dismantled. The resin gels were immersed in 1 ml of nitric acid (1 mol L^−1^) for 24 h. The Cd concentration in the eluent was measured using graphite furnace atomic absorption spectrometry (GFAAS; Hitachi z-2000, Japan). The detection limit and precision of the GFAAS were 0.01 μg L^−1^ and 0.05 μg L^−1^ < 3%, respectively. The detection limits of the GFAAS have also been reported by other studies. Tüzen^49^ reported that the detection limit for the GFAAS method was 0.065 μg·L^−1^ for Cd, and Bakırdere *et al*.^50^ reported that the limit of detection (LOD) for Cd by GFAAS was 0.036 μg·L^−1^. The average concentration of the solution blanks (including the matrix of 1 M HNO_3_) was 0.348 ± 0.021 μg·L^−1^. These concentrations were measurable by this instrument and within the previously reported concentration ranges.

The DGT-measured Cd concentration (*C*_DGT_) was calculated using Equation (1)^a^.

1 $${{C}}_{{\rm{DGT}}}={\rm{M}}{\rm{\Delta }}{\rm{g}}/({\rm{DAt}})$$

^a^M is the accumulated mass of Cd (μg); Δg is the thickness of the diffusive layer (cm); D is the diffusion coefficient of Cd in the diffusive layer (cm^2^·s^−1^); A is the area of the DGT exposure window (cm^2^); and t is the DGT deployment time (s)^5,48^.

### Soil solution and single-step extraction methods

After retrieval of the DGT devices, all soil samples were transferred to 50 ml polyethylene tubes and centrifuged at 5000 r·min^−1^ for 15 min to extract the soil solution^39^. Three widely used traditional extractants were used: 0.05 mol·L^−1^ EDTA, 0.01 mol·L^−1^ CaCl_2_, and 0.11 mol·L^−1^ HAc. The extraction procedures are shown in Table 4. The extractants were acidified using HNO_3_ and measured via AAS and GFAAS.

**Table 4:** Procedures for the single-step extraction methods used in this study.

Extractants	Procedure	References
EDTA	2.0 g of soil was extracted with 20 ml of 0.05 mol·L^−1^ EDTA, and the pH (7.0) was adjusted with ammonia solution; the mixture was shaken for 2 h	(Wear and Evans, 1968)^51^
HAc	0.5 g of soil was extracted with 20 ml of 0.11 mol·L^−1^ HAc and shaken for at least 16 h (overnight)	(Houba *et al*., 1996)^52^
CaCl_2_	2.0 g of soil was extracted with 20 ml of 0.01 mol·L^−1^ CaCl_2_ and shaken for 3 h	(Novozamsky *et al*., 1993)^53^

### Statistical analysis

A stepwise multiple regression analysis was performed using SPSS 23.0. The plant biomass and plant Cd content data were tested using Duncan’s Multiple Range Test (DMRT) at 5% to identify significant differences among the treatments. Pearson’s correlation coefficient was used to assess the relationships between various bioavailable indicators of Cd uptake by plants and the available forms in soils measured by different methods. All figures were generated using SigmaPlot 12.5.
